# Relevance of HLA-DP/DQ and INF-λ4 Polymorphisms to COVID-19 Outcomes

**DOI:** 10.3389/bjbs.2023.11044

**Published:** 2023-01-20

**Authors:** Amany A. Ghazy, Abdullah N. Alrasheedi, Mohammed Elashri, Hany Hussein Moussa, Eman K. Rashwan, Ibrahim Amer, Shimaa El Sharawy, Shimaa Elgamal, Salwa Tawfik, Mohamed Abdelnasser, Amel Elsheredy

**Affiliations:** ^1^ Department of Pathology, Microbiology and Immunology Division, College of Medicine, Jouf University, Sakaka, Saudi Arabia; ^2^ Department of Otolaryngology - Head and Neck Surgery, College of Medicine, Jouf University, Sakaka, Aljouf, Saudi Arabia; ^3^ Department of Ophthalmology, Faculty of Medicine, Kafrelsheikh University, Kafrelsheikh, Egypt; ^4^ Department of Chest Disease, Faculty of Medicine, Kafrelsheikh University, Kafrelsheikh, Egypt; ^5^ Department of Physiology, College of Medicine, Jouf University, Sakaka, Saudi Arabia; ^6^ Department of Hepatology, Gastroenterology and Infectious Diseases, Faculty of Medicine, Kafrelsheikh University, Kafrelsheikh, Egypt; ^7^ Department of Tropical Medicine and Infectious Diseases, Faculty of Medicine, Tanta University, Tanta, Egypt; ^8^ Department of Neurology, Faculty of Medicine, Kafrelsheikh University, Kafrelsheikh, Egypt; ^9^ Department of Internal Medicine, National Research Center, Cairo, Egypt; ^10^ Faculty of Medicine, Kafrelsheikh University, Kafrelsheikh, Egypt; ^11^ Microbiology Department, Medical Research Institute, Alexandria University, Alexandria, Egypt

**Keywords:** genetic polymorphism, HLA, IFNλ4, COVID-19 outcomes, prognostic markers

## Abstract

**Background:** Single nucleotide polymorphisms provide information on individuals’ potential reactions to environmental factors, infections, diseases, as well as various therapies. A study on SNPs that influence SARS-CoV-2 susceptibility and severity may provide a predictive tool for COVID-19 outcomes and improve the customized coronavirus treatment.

**Aim:** To evaluate the role of human leukocyte antigens DP/DQ and IFNλ4 polymorphisms on COVID-19 outcomes among Egyptian patients.

**Participants and Methods:** The study involved 80 patients with severe COVID-19, 80 patients with mild COVID-19, and 80 non-infected healthy volunteers. Genotyping and allelic discrimination of HLA-DPrs3077 (G/A), HLA-DQrs7453920 (A/G), and IFNλ4 rs73555604 (C/T) SNPs were performed using real-time PCR.

**Results:** Ages were 47.9 ± 8, 44.1 ± 12.1, and 45.8 ± 10 years in severe, mild and non-infected persons. There was a statistically significant association between severe COVID-19 and male gender (*p* = 0.002). A statistically significant increase in the frequency of HLA-DPrs3077G, HLA-DQrs7453920A, and IFNλ4rs73555604C alleles among severe COVID-19 patients when compared with other groups (*p* < 0.001). Coexistence of these alleles in the same individual increases the susceptibility to severe COVID-19 by many folds (*p* < 0.001). Univariate and multivariate logistic regression analysis for the studied parameters showed that old age, male gender, non-vaccination, HLA-DQ rs7453920AG+AA, HLA-DPrs3077GA+GG, and IFNλ4rs73555604CT+CC genotypes are independent risk factors for severe COVID-19 among Egyptian patients.

**Conclusion**: HLA-DQ rs7453920A, HLA-DPrs3077G, and IFNλ4rs73555604C alleles could be used as markers of COVID-19 severity.

## Background

Severe acute respiratory syndrome coronavirus-2 (SARS-CoV-2) infection causes Coronavirus disease 2019 (COVID-19) which is deliberated as a global public health crisis. SARS-CoV-2 is an emerging, enveloped, highly infectious, positive sense RNA virus (1).

In spite of the same mode of transmission and pathogenesis of SARS-CoV-2, the global distribution and outcomes of COVID-19 show remarkable differences among the different countries (2). Recent genomic studies revealed a possible link between these disparities and polymorphisms in genes involved in viral entry into cells, antigen presentation to immune system and the immune response (3–6).

For any viral infection, viral antigen presentation and induction of cellular immunity occurs mainly through human leukocyte antigen (HLA). Recently, we have studied the role of HLA-DP/DQ polymorphisms in many diseases as ovarian cancer (7), hepatitis C virus (HCV) infection and response to treatment (8), Prostate cancer (PCa) (9), *Helicobacter pylori* recurrence (10), and type 1 diabetes mellitus (T1DM) (11). Results of these studies showed variable results; for example, HLA-DPrs3077G allele was more frequent among PCa patients than the control group (9). However, HLA-DPrs3077A allele was associated with higher risk to ovarian cancer (7), HCV (8), *H. pylori* infection (10), and T1DM (11). In addition, coexistence of HLA-DPrs3077A and HLA-DQrs7453920A alleles in the same person increases the risk of T1DM by seven times (11).

Furthermore, iterferon-lambda-4 (IFNλ4), member in type III interferons (IFNIII), is considered a functional gene in more than 95% of the African population. It controls the expression of the antiviral IFN-stimulated gene-5 (ISG-5). IFNλ4 gene polymorphism will affect the expression levels of IFNλ4, ISGs, and in turn the antiviral response of IFN-α (12). We have studied the effect of IFNλ4rs73555604 polymorphism on HCV response to treatment and found that 75% of non-responders carry IFNλ4rs73555604T allele, and persons carrying T allele have 2.5 fold higher odds ratio to be non-responders to treatment (12). Thus, both HLA and IFNλ4 genetic variations could have a great impact on SARS-CoV-2 pathogenesis and in turn COVID-19 outcome.

COVID-19 unpredictable outcomes, even among young adults, have raised the question of how genetic susceptibility could explain these variations (13). This highly infective SARS-CoV-2 virus showed very wide dissemination globally with more than 27 million infections (14).

At the beginning of the pandemic, researchers tend to develop an effective vaccine that focuses on SARS-CoV-2 Spike protein. Later, it was reported that vaccines that induce a T cell response to other viral proteins could be more efficient, especially with the wide variation of COVID-19 clinical outcomes and severity where some patients deteriorate to the fatal SARS while others remain asymptomatic or have mild symptoms (14). Thus studying of the factors affecting the innate response to SARS-CoV-2, as INF, and factors initiating T cell response as HLA could be helpful. Genetic factors may have a role in the increased SARS-CoV-2 transmission, augmentation of the cytokine storm, delayed immune regulation, and variable COVID-19 outcomes. However, these aspects remain unclear. Thus the current study aims to assess the influence of HLA-DPrs3077 (G/A), HLA-DQ rs7453920 (A/G), IFNλ4 rs73555604 (C/T) on the outcomes of COVID-19.

## Subjects and Methods

### Study Design

The study involved 240 individuals divided into three groups: group 1) 80 patients with severe COVID-19 infection; group 2) 80 patients with mild COVID-19 infection; and group 3) included 80 age- and sex-matched non-infected persons. Inclusion criteria for COVID-19 patients were positive SARS-CoV-2 RT-PCR test of nasopharyngeal or oropharyngeal swabs and clinical symptoms of COVID-19. Clinical data include age, sex, co-morbidity (as diabetes mellitus, hypertension, bronchial asthma) and symptoms (fever, loss of smell, cough, loss of taste, sore throat, headache, dyspnea, muscle ache, and/or diarrhea) (13). Severity of COVID-19 infection was judged as follows: 1) mild cases with no pneumonia on lung CT, mild clinical symptoms, and O2 saturation >93%; 2) severe cases with respiratory rate >30/minutes, severe clinical symptoms, and O2 saturation ≤93% at rest (15). Level of consciousness was determined by Glasgow Coma Scale. Exclusion criteria were autoimmune diseases, hepatic, cardiac, and/or renal decompensation. Non-infected persons showed negative SARS-CoV-2 RT-PCR test of nasopharyngeal or oropharyngeal swabs and did not get infected before. Eye examination was done by direct ophthalmoscope +/- cobalt blue light and fluorescein stain strips. All participants were asked about receiving COVID-19 vaccine and doses received.

### Ethics Approval

Ethics Review Committee of the Faculty of Medicine, Kafrelskeikh University, Egypt has approved the study protocol (MKSU 10-12-20). The ethical guidelines of the 1975 Declaration of Helsinki were followed without any risk to the participants. Informed written consents were gathered from participants.

### Samples

Four ml of venous blood was drawn from all the subjects for DNA extraction and genotyping.

### Genotyping of HLA-DQ rs7453920 (A/G), HLA-DPrs3077 (G/A) and IFNλ4 rs73555604 (C/T) SNP

The genomic DNA of all participants was extracted from the venous blood samples using QIAamp DNA Blood Mini Kit (https://www.qiagen.com/us/products/discovery-and-translational- research/dna-rna-purification/dna-purification/genomic-dna/qiaamp-dna-blood-kits). Genotyping of HLA-DQ/DP, and IFNλ4 rs73555604 SNPs were done using TaqMan dual-labeled probes, as described by Ghazy et al. (7), and Abu El Nazar et al. (12), respectively. In brief, 12.5 μl TaqMan universal PCR master mix, 1.25 μl TagMan SNP genotyping assay (probes), 9.25 μl DNase-free water and 2 μl genomic DNA were mixed together in PCR tubes and put in StepOne™ Real-Time PCR System (Applied Biosystems, Life Technologies, United States). The thermal profile was programmed as follow: 95°C for 10 min (initial denaturation), 40 cycles of 92°C for 15 s (denaturation) and 60°C for 1 min (annealing/extension), then hold at 4°C. Genotypes were determined by fluorescence signal increase when the probes hybridized to the complementary sequence in tested samples and cleaved from the dye. A significant increase in FAM dye only indicates homozygosity for the wild allele (second allele), in VIC dye only means homozygosity for the mutant allele (first allele) and if both fluorescence signals increase, this indicates heterozygosity (7, 12)**.** In each run, samples without DNA were used as negative controls and three samples were repeated to ensure reproducibility.

### Statistical Analysis of the Data

Data were analyzed using IBM SPSS software package version 20.0 (Armonk, NY, United States: IBM Corp). Kolmogorov- Smirnov test was used to verify the normality of variables distribution. The equilibrium of the studied samples was explored using the Hardy-Weinberg equation. Qualitative data were expressed as numbers and percent. Quantitative data were expressed by median, mean, and standard deviation (SD). Comparisons between groups were performed using the chi-squared test, Student’s t-test, and F-test (ANOVA). Odds ratios (OR) with 95% confidence intervals (CI) were used to calculate the ratio of the odds of an event in the risk group to the non-risk group. Statistical significance was set at 5%.

## Results

### Subjects’ Demographic Data

The study involved 80 patients with severe COVID-19 (57.5% males, 42.5% females), 80 patients with mild COVID-19 infection (33.8% males, 66.2% females), and 80 non-infected persons (33.8% males, 66.2% females). Ages were ranging between 21 and 67 years in all groups with mean ± SD equals 47.9 ± 8, 44.1 ± 12.1, and 45.8 ± 10 years for severe, mild, and non-infected groups, respectively. No statistically significant difference was found between the groups in terms of age (*p* = 0.055). However, there was a statistically significant association between severe COVID-19 and male gender (*p* = 0.002). Regarding eye manifestations, 40% of severe COVID-19 patients showed ocular manifestations as red eye and discharge. However, the COVID-19-specific assaults such as occlusion of the central retinal artery or vein, sub-conjunctival hemorrhage, photophobia, and/or lid edema were not statistically significant (*p* < 0.06).

Regarding the vaccination status, all non-infected persons were vaccinated against COVID-19 while 51.3% and 91.3% of severe and mild COVID-19 patients have received the two doses of vaccine (*p* < 0.001).

### Genotypic and Allelic Determination

Hardy-Weinberg equation (HWE) was used to test for any significant differences between the observed and expected genotype frequencies in the studied groups. Results revealed the absence of any statistically significant differences in the studied groups (Table 1).

**TABLE 1:** Hardy-Weinberg for different SNPs in each group.

	Non-infected (*n* = 80)	Mild COVID-19 (*n* = 80)	Severe COVID-19 (*n* = 80)
HLA-DQrs7453920 (A/G)			
GG^®^	32 (40%)	27 (33.8%)	11 (13.8%)
AG	43 (53.8%)	45 (56.3%)	27 (33.8%)
AA	5 (6.3%)	8 (10%)	42 (52.5%)
^HW^p	*p* = 0.057	*p* = 0.086	*p* = 0.066
HLA-DPrs3077 (G/A)			
AA^®^	16 (20%)	13 (16.3%)	2 (2.5%)
GA	48 (60%)	40 (50%)	10 (12.5%)
GG	16 (20%)	27 (33.8%)	68 (85%)
^HW^p	*p* = 0.074	*p* = 0.778	*p* = 0.052
IFNλ4rs73555604 (C/T)			
TT^®^	24 (30.0%)	20 (25%)	7 (8.8%)
CT	41 (51.3%)	45 (56.3%)	23 (28.8%)
CC	15 (18.8%)	15 (18.8%)	50 (62.5%)
^HW^p	*p* = 0.733	*p* = 0.247	*p* = 0.087

^a^ Statistically significant at *p* ≤ 0.05.

HW, Hardy-Weinberg equilibrium; ^®^, Reference group; *p*, *p*-value for Chi square for goodness of fit.

HLA-DPrs3077 (G/A) genotyping in the studied samples showed that all genotypes are expressed in all groups. However, HLA-DP rs3077GG Genotype frequency is markedly increased among patients with severe COVID-19 in comparison with the other groups (*p* < 0.001). At the allele level, the frequency HLA-DP rs3077G allele among patients with severe COVID-19 is significantly higher than mild and non-infected groups (*p* < 0.001) (Table 2). OR (95% CI) of 7.3 (3.9–13.8) appeared when the frequency of G allele among severe COVID-19 patients was compared with the mild cases and 10.4 (5.6–19.6) when compared with the controls (Table 3).

**TABLE 2:** Comparison between the three studied groups according to different SNPs.

SNPs	Non-infected (*n* = 80)	Mild COVID-19 (*n* = 80)	Severe COVID-19 (*n* = 80)	Sig. bet. groups
HLA-DQrs7453920 (A/G)				
GG^®^	32 (40%)	27 (33.8%)	11 (13.8%)	*p*_1_ = 0.559
AG	43 (53.8%)	45 (56.3%)	27 (33.8%)	*p*_2_ < 0.001^a^
AA	5 (6.3%)	8 (10%)	42 (52.5%)	*p*_3_ < 0.001^a^
Allele				*p*_1_ = 0.350
G^®^	107 (66.9%)	99 (61.9%)	49 (30.6%)	*p*_2_ < 0.001^a^
A	53 (33.1%)	61 (38.1%)	111 (69.4%)	*p*_3_ < 0.001^a^
HLA-DPrs3077 (G/A)				
AA^®^	16 (20%)	13 (16.3%)	2 (2.5%)	*p*_1_ = 0.146
GA	48 (60%)	40 (50%)	10 (12.5%)	*p*_2_ < 0.001^a^
GG	16 (20%)	27 (33.8%)	68 (85%)	*p*_3_ < 0.001^a^
Allele				*p*_1_ = 0.116
A^®^	80 (50%)	66 (41.3%)	14 (8.8%)	*p*_2_ < 0.001^a^
G	80 (50%)	94 (58.8%)	146 (91.3%)	*p*_3_ < 0.001^a^
IFNλ4rs73555604 (C/T)				
TT^®^	24 (30.0%)	20 (25%)	7 (8.8%)	*p*_1_ = 0.760
CT	41 (51.3%)	45 (56.3%)	23 (28.8%)	*p*_2_ < 0.001^a^
CC	15 (18.8%)	15 (18.8%)	50 (62.5%)	*p*_3_ < 0.001^a^
Allele				*p*_1_ = 0.653
T^®^	89 (55.6%)	85 (53.1%)	37 (23.1%)	*p*_2_ < 0.001^a^
C	71 (44.4%)	75 (46.9%)	123 (76.9%)	*p*_3_ < 0.001^a^

^a^ Statistically significant at *p* ≤ 0.05.

*p*_1_, *p*-value for Chi square test for comparing between non-infected and mild COVID-19; *p*_2_, *p*-value for Chi square test for comparing between non-infected and severe COVID-19; *p*_3_, *p*-value for Chi square test for comparing between mild COVID-19 and severe COVID-19.

**TABLE 3:** Univariate analysis for severe cases regarding to different SNPs genotype and allele.

SNPs genotypes and alleles	Severe vs. mild^®^		Severe vs. control^®^	
	*p*	OR (LL–UL 95% CI)	*p*	OR (LL–UL 95% CI)
HLA-DQrs7453920 (A/G)				
GG^®^		1.000		1.000
AG	0.371	1.5 (0.6–3.4)	0.158	1.8 (0.8–4.2)
AA	<0.001^a^	12.9 (4.6–36.1)	<0.001^a^	24.4 (7.7–77.4)
Allele				
G^®^		1.000		1.000
A	<0.001^a^	3.7 (2.3–5.8)	<0.001^a^	4.6 (2.9–7.3)
HLA-DPrs3077 (G/A)				
AA^®^		1.000		1.000
GA	0.562	1.6 (0.3–8.4)	0.537	1.7 (0.3–8.4)
GG	<0.001^a^	16.4 (3.5–77.4)	<0.001^a^	34 (7.1–163)
Allele				
A^®^		1.000		1.000
G	<0.001^a^	7.3 (3.9–13.8)	<0.001^a^	10.4 (5.56–19.6)
IFNλ4rs73555604 (C/T)				
TT^®^		1.000		1.000
CT	0.456	1.5 (0.5–4)	0.193	1.9 (0.7–5.2)
CC	<0.001^a^	9.5 (3.4–26.8)	<0.001^a^	11.4 (4.1–31.7)
Allele				
T^®^		1.000		1.000
C	<0.001^a^	3.8 (2.3–6.1)	<0.001^a^	4.2 (2.6–6.8)

^a^ Statistically significant at *p* ≤ 0.05.

OR, Odd’s ratio; ^®^, Reference group; CI, confidence interval; LL, lower limit; UL, upper limit; *p*, *p*-value for univariate regression analysis for comparing with the reference genotype.

With respect to the HLA-DQ rs7453920 (A/G) SNP, three genotypes (GG, GA, and AA) were distributed in all groups with a higher frequency of the AA genotype among patients with severe COVID-19 in comparison with the mild and control groups (*p* < 0.001). At the allele level, the frequency HLA-DQ rs7453920A allele among patients with severe COVID-19 is significantly higher than the mild and negative controls (*p* < 0.001) (Table 2). Univariate analysis of the studied alleles declared that OR (95% CI) of HLA-DQ rs7453920A allele was 12.9 (4.6–36.1) when severe COVID-19 patients compared with the mild group and 24.4 (7.7–77.4) when compared with the controls (Table 3).

Genotyping of IFNλ4rs73555604 (C/T) SNP showed that CC genotype was more frequent among severe COVID-19 patients when compared with the mild cases (62.5% vs. 18.8%) and the negative controls (62.5% vs. 18.8%) (*p* < 0.001). The same for C allele which show statistically significant increase among severe COVID-19 patients (67.5%) when compared with the mild and non-infected groups (39.4%, 49.4%, respectively) (*p* < 0.001) (Table 2). Univariate analysis of the studied alleles declared that OR (95% CI) IFNλ4rs73555604 C was 3.8 (2.3–6.1) (*p* < 0.001) when severe COVID-19 patients compared with the mild cases and 4.2 (2.6–6.8) (*p* < 0.001) when compared with the controls (Table 3).

Statistical analysis of for HLA-DQ & HLA-DP & IFNλ4 in the three study groups clarified that the presence of HLA-DQ rs7453920A, HLA-DP rs3077G, IFNλ4rs73555604C in the same person increased the risk of severe COVID-19 manifestation markedly that may reach hundred times (Table 4).

**TABLE 4:** Haplotype frequency for HLA-DQ & HLA-DP & IFNλ4 in the three study groups.

HLA-DQ & HLA-DP & IFNλ4	Haplotype frequencies (%)			Severe vs. mild^®^		Severe vs. non-infected^®^	
	Non-infected (*n* = 160)	Mild COVID-19 (*n* = 160)	Severe COVID-19 (*n* = 160)	*p*_1_	OR (95% CI) (LL–UL)	*p*_2_	OR (95% CI) (LL–UL)
GAT^®^	50 (31.3%)	40 (25%)	1 (0.6%)		1.000		1.000
GAC	15 (9.4%)	11 (6.9%)	5 (3.1%)	0.011^a^	18.2 (1.9–172.2)	0.013^a^	16.7 (1.8–154)
GGT	19 (11.9%)	26 (16.3%)	14 (8.8%)	0.004^a^	21.6 (2.7–173.8)	0.001^a^	36.8 (4.5–299.8)
GGC	23 (14.4%)	22 (13.8%)	29 (18.1%)	<0.001^a^	52.7 (6.7–413.8)	<0.001^a^	63. (8.1–491.5)
AAT	7 (4.4%)	7 (4.4%)	5 (3.1%)	0.004^a^	28.6 (2.9–282.8)	0.002^a^	35.7 (3.6–352)
AAC	8 (5%)	8 (5%)	3 (1.9%)	0.026^a^	15 (1.4–163.2)	0.016^a^	18.8 (1.7–203.2)
AGT	13 (8.1%)	12 (7.5%)	17 (10.6%)	<0.001^a^	56.7 (6.8–470.9)	<0.001^a^	65.4 (8–537.7)
AGC	25 (15.6%)	34 (21.3%)	86 (53.8%)	<0.001^a^	101.2 (13.4–765.5)	<0.001^a^	172 (22.6–1308.2)

^a^ Statistically significant at *p* ≤ 0.05.

^®^, reference group; OR, odds ratio; CI, confidence interval; LL, lower limit; UL, upper limit; *p*_1_, *p*-value for comparing between severe from mild COVID-19; *p*_2_, *p*-value for comparing between severe COVID-19 and non-infected persons.

**FIGURE 1:**
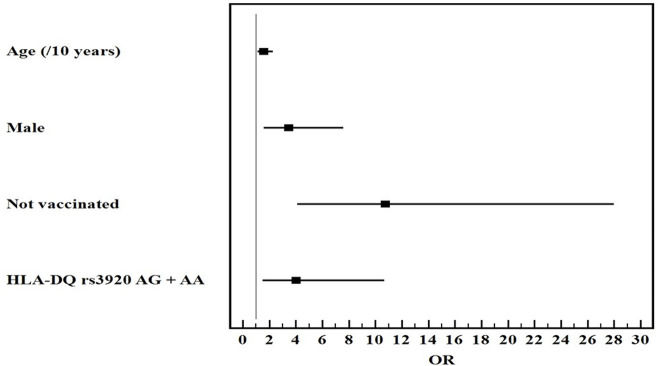
Multivariate logistic regression analysis for the parameters affecting severe from mild COVID-19 (*n* = 80 vs. 80). OR (LL–UL 95% CI) for age (/10 years) is 1.6 (1.1–2.3) (*p* = 0.013), male gender is 3.5 (1.6–7.6) (*p* = 0.002), Not vaccinated patients is 10.8 (4.1–28) (*p* < 0.001), and HLA-DQ rs7453920AG+AA genotypes is 4 (1.5–10.7) (*p* = 0.005).

**FIGURE 2:**
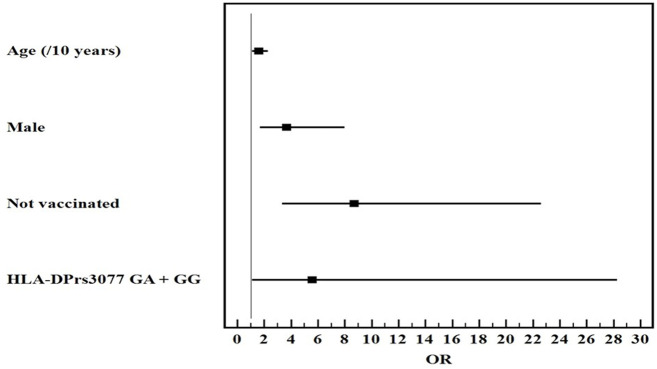
Multivariate logistic regression analysis for the parameters affecting severe from mild COVID-19 (*n* = 80 vs. 80). OR (LL–UL 95% CI) for age (/10 years) is 1.6 (1.1–2.3) (*p* = 0.011), male gender is 3.7 (1.7–8) (*p* = 0.001*), Not vaccinated patients is 8.7 (3.4–22.6) (*p* < 0.001), and HLA-DPrs3077GA+GG genotypes is 5.6 (1.1–28.3) (*p* = 0.038).

**FIGURE 3:**
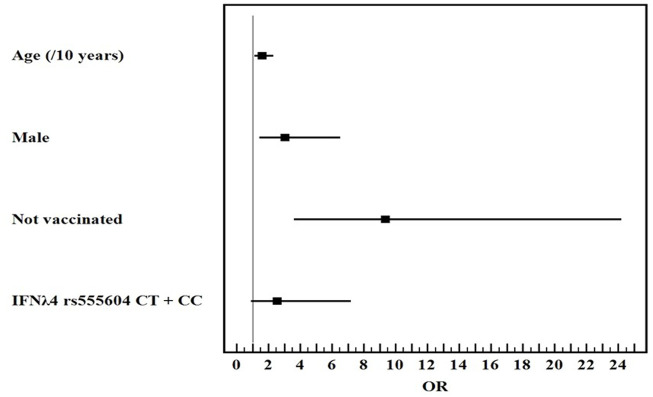
Multivariate logistic regression analysis for the parameters affecting severe from mild COVID-19 (*n* = 80 vs. 80). OR (LL–UL 95% CI) for age (/10 years) is 1.6 (1.1–2.3) (*p* = 0.008), male gender is 3.1 (1.4–6.5) (*p* = 0.004), Not vaccinated patients is 9.4 (3.6–24.2) (*p* < 0.001), and IFNλ4 rs73555604CT+CC genotypes is 2.6 (0.9–7.2) (*p* = 0.077).

Univariate and multivariate logistic regression analysis for the studied parameters affecting severe from mild COVID-19 infection showed that old age, male gender, non-vaccination, HLA-DQ rs7453920AG+AA, HLA-DPrs3077GA+GG, and IFNλ4 rs73555604CT+CC genotypes are independent risk factors for the deterioration of COVID-19 manifestations (Figures 1–3).

## Discussion

COVID-19 is an acute respiratory disease caused by SARS-CoV2 infection and has a wide range of clinical manifestations and severity; ranging from asymptomatic carriers and mild flu-like symptoms to severe respiratory distress and death (5, 6, 16).

Single nucleotide polymorphisms (SNPs) provide information on individuals’ potential reactions to various infections, environmental factors, diseases, as well as therapies (17). Since SNP prevalence differ among demographic groups, analysis of SNPs that influence SARS-CoV-2 susceptibility and severity may provide a predictive tool for COVID-19 outcomes and improve the customized coronavirus treatment, particularly among the high risk groups (18).

Regarding the biology of SARS-CoV-2, there are several factors that influence its pathogenesis as binding of the viral spike (S) protein to angiotensin-converting enzyme 2 (ACE2) receptors, priming of viral proteins by host cell proteases as transmembrane protease serine 2 (TMPRSS2), interferon-induced transmembrane protein 3 (IFITM3), viral antigens presentation on HLA, cytokines. SNPs in ACE2, TMPRSS2, cytokines, and vitamin D receptor (VDR). SNP in genes controlling these factors could have an impact on COVID-19 incidence and severity (19–22).

To manage COVID-19 effectively, it is critical to comprehend the variations that could affect the disease’s severity generally and at the populations levels (6). Thus performing genetic analysis to detect genetic variants that affect viral pathogenesis could help in risk stratification of patients to plan their management accordingly.

Recently published systematic review and meta-analysis on the relation between ACE1, ACE2, TMPRSS2, IFITM3, and VDR genes polymorphisms and COVID-19 outcomes has elucidated the potential involvement of these SNPs in COVID-19 outcomes. They stated that ACE1rs1799752 minor allele I and ACE2rs2285666A are associated with decreased COVID-19 severity while TMPRSS2 rs12329760 is linked to severe COVID-19 outcomes. No association was found between IFITM3 rs12252 and VDR polymorphisms with disease severity (22). However, genes involved in the regulation of viral antigens recognition, presentation, and antiviral immune response should be considered also as candidates for genetic association studies on COVID-19 severity in different ethnic groups. Thus the current case-control study has assessed the influence of HLA-DP/DQ and IFNλ4 polymorphisms on COVID-19 outcomes among Egyptian patients.

The study enrolled 240 individuals and genotyping of HLA DPrs3077 (G/A), HLA-DQ rs7453920 (A/G), and IFNλ4 rs73555604 (C/T) SNPs was performed using real-time PCR. Results declared a statistically significant association between severe COVID-19 and male gender (*p* = 0.002). HLA-DPrs3077GG, HLA-DQrs7453920AA genotypes frequencies were higher among severe COVID-19 patients in comparison with the mild and non-infected groups. The same was confirmed at the allele level where the frequency of HLA-DPrs3077G, HLA-DQrs7453920A were markedly increased among severe COVID-19 patients in comparison with the other groups. These genetic variants may have impacts on the host immune response against SARS-CoV-2 and in-turn COVID-19 outcome. This agrees with other researchers who reported that HLA class I and II genetic variants are implicated in the modulation of immune response to diverse viral antigens (23, 24).

Kachuri et al. (3) have stated that HLA-II controls the landscape of antiviral humoral immune response. They identified 40 independent loci and 14 independent alleles, 7 of which exhibited pleiotropic effects across viral families. They have conducted conditional analyses of 38,655 SNPs in the HLA regional to identify the statistically independent SNP or classical HLA allele that could influence the humoral immune response to common viral antigens. A significant association was observed between HLA-DQB1 rs9273325GT/G and its expression on T-helper 2 (Th) lymphocytes, also, rs1130420 influenced the expression of 8 HLA-II genes in naïve B cells and Th17 cell. They concluded that HLA-II genes have an essential role in modulating IgG levels against viral infection. They considered HLA-DRB1 and -DQB1 as crucial genetic factors governing host vulnerability to viral infections. Another study conducted on Italian patients suffering from COVID-19 has found a strong correlation between DQB1*06:02 allele and severe COVID-19 (25). A study performed on infected Brazilian couples (one was symptomatic while the partner remained asymptomatic) using exome sequencing and variant calling across the highly polymorphic HLA region. They observed an association between DOB*01:02 with symptomatic SARS-CoV2 infections (5).

Shah et al. (4) have reported that SARS-CoV peptides are presented with both HLA-I and -II to activate CD8^+^ cytotoxic T-lymphocytes (CTLs) and CD4^+^ T cells, respectively. This promotes antibody production, cytokine release and cytolytic activity of CTL. HLA polymorphism enables the presentation of some T cell epitopes very well over others. Some MHC alleles are associated with downregulated HLA expression which in turn has been correlated with disease severity. Zhao et al. (26) have reported that after recovery, strong immune responses are induced by generating a memory T-lymphocytes against SARS-CoV and MERS-CoV. Reactivation of these memory T cells results in local damage due to cross-reactivity.

Regarding genotyping of IFNλ4 rs73555604 (C/T) SNP, results showed a statistically significant increase of IFNλ4rs73555604C alleles frequency among severe COVID-19 patients in comparison with the mild and non-infected groups. It is known that upon viral entry innate immunity recognizes the foreign viral particles, synthesizes IFN, and initiates a signaling cascade to restrict virus transmission to adjacent cells (27). IFNs play an essential role in limiting virus spread, promoting macrophage phagocytosis of antigens, as well as natural killer (NK) cells restriction of infected target cells. Thus, polymorphisms in IFNs genes could affect its expression, immune response, and viral survival in the host cells (28).

It was reported that IFN-λ is secreted by Plasmacytoid dendritic cells (pDC) that respond to this cytokine in an autocrine manner to surge the anti-viral responses (29). Moreover, Wellington et al. (30) have found that IFN-λ induce IFITM3 expression in pDC cells to a level similar to induction by type I IFN. IFITM3 produces an immune effector protein that promotes mucosal immune cell durability by increasing CD8+CTL number in the airways, which is vital in immunity against viral diseases. This protein is also involved in viral invasion and linked to the COVID‐19 severity and the cytokine storm (6). Zhang et al. (31) have noticed that IFITM3rs12252C allele is associated with severe COVID-19 illness too.

Additional research has reviewed the studies exploring connection between HLA genotypes and individual responses to SARS-CoV-2 infection. They noticed that, in almost all studies, HLA polymorphisms may influence the vulnerability, progression and severity of COVID-19. They explained this by the crucial role of HLA molecules in the presentation of viral peptide, and activation of both humoral and cellular anti-viral immune response (32). Furthermore, the huge molecular variability of HLA alleles among the human populations could be accountable for the different rates of SARS-CoV-2 infection and different outcomes of COVID-19.

Langton et al. (33) have provide an evidence that HLA genotype affects COVID-19 outcomes among Europeans. They found that DRB1*04:01 significantly affects the severity of COVID-19 and it is more frequent among the North Western European population. In addition, frequency of DQA1*01:01-DQB1*05:01-DRB1*01:01 haplotype was decreased in the asymptomatic persons.

A study was performed by Hu et al. (34) on patients with white British ancestry to identify genetic variants that could affect the pathogenesis of COVID-19. They found that COVID-19 mortality increased markedly among the patients with 8 genetic variants that affect functions of cilia, mitochondria, cardiovascular system, agglutination, and immune system during SARS-CoV-2 infection. The concluded that genetic modifications have a role in COVID-19 heterogeneous susceptibility, and may have a potential impact on the therapeutic regimens.

Dieter et al. (35) have performed a meta-analysis and system in review to identify genetic polymorphisms related to COVID-19 pathogenesis. They recognized that some genetic mutations were linked to protection against COVID-19 (as HLA-A*30 and *CCR5* rs333Del), others could be risk factors as *APOE* rs429358C allele or deteriorate the outcome of COVID-19 as HLA-B*38, HLA-C*6, and *ApoE* rs429358C alleles were associated with risk for severe forms of COVID-19.

Another interesting finding is that coexistence of HLA-DPrs3077G, HLA-DQrs7453920A, and IFNλ4rs73555604C alleles in the same individual increases the susceptibility to severe COVID-19 by several folds. Univariate and multivariate logistic regression analysis for the studied parameters showed that old age, male gender, non-vaccination, HLA-DQrs7453920AG+AA, HLA-DPrs3077GA+GG, and IFNλ4rs73555604CT+CC genotypes are independent risk factors for the deterioration of COVID-19 manifestations. These results ensure the multifactorial nature of COVID-19 severity. This agrees with Adli et al. (6) who stated that gender and age differences, genetic factors and comorbid diseases contribute to variation in COVID-19 presentation and a higher risk of mortality in certain ethnic groups.

All of this may shed light on the role of HLA-DP/DQ polymorphisms as genetic modulators in pathogenesis and outcomes of SARS-CoV-2 infection. However, the main limitations in the current study is the small sample size, and lack of comparison with serum lab markers, virus strain, and the effects of drug treatments on outcomes and severity. Thus further research on larger scale including more comparative parameters are recommended.

## Conclusion

HLA-DQ rs7453920A, HLA-DP rs3077G, and IFNλ4rs73555604C alleles could be reliable markers for COVID-19 severity and outcomes.

## Summary Table

### What Is Known About This Subject

• COVID-19 unpredictable outcomes, even among young adults, have raised the question of how genetic susceptibility could explain these variations.

### What This Paper Adds

• Genetic polymorphisms of HLA-DPrs3077 (G/A), HLA-DQrs7453920 (A/G), IFNλ4 rs73555604 (C/T) influence the outcomes of COVID-19 and some alleles are associated with severe manifestations.
• This work represents an advance in biomedical science because presence of HLA-DQ rs7453920A, HLA-DPrs3077G, and IFNλ4rs73555604C alleles are associated with higher risk of severe COVID-19 outcome, particularly if existed together. They could be used as markers of COVID-19 severity.

## Data Availability

The raw data supporting the conclusion of this article will be made available by the authors, without undue reservation.

## Abbreviations

ACE2, Angiotensin-converting enzyme 2; COVID-19, coronavirus disease 2019; CTLs, cytotoxic T-lymphocytes; HLA, human leukocyte antigen; HWE, Hardy-Weinberg equation; IFITM3, interferon-induced transmembrane protein 3; IFNλ4, Interferon-lambda-4; ISG-5, IFN-stimulated gene-5; NK cells, natural killer cells; pDC, plasmacytoid dendritic cells; SARS-CoV-2, severe acute respiratory syndrome coronavirus 2; SNP, Single nucleotide polymorphism; TMPRSS2, Transmembrane protease serine 2; VDR, vitamin D receptor.
